# Fungal secretome profile categorization of CAZymes by function and family corresponds to fungal phylogeny and taxonomy: Example *Aspergillus* and *Penicillium*

**DOI:** 10.1038/s41598-020-61907-1

**Published:** 2020-03-20

**Authors:** Kristian Barrett, Kristian Jensen, Anne S. Meyer, Jens C. Frisvad, Lene Lange

**Affiliations:** 1 Department for Biotechnology and Biomedicine, Building 221, Technical University of Denmark, DK-2800 Lyngby, Denmark; 2 The Novo Nordisk Foundation Center for Biosustainability, Building 220, Technical University of Denmark, DK-2800 Lyngby, Denmark; 3 LLa Bioeconomy, Research & Advisory, Karensgade 5, DK-2500 Valby, Denmark

**Keywords:** Biochemistry, Ecology, Evolution, Microbiology

## Abstract

Fungi secrete an array of carbohydrate-active enzymes (CAZymes), reflecting their specialized habitat-related substrate utilization. Despite its importance for fitness, enzyme secretome composition is not used in fungal classification, since an overarching relationship between CAZyme profiles and fungal phylogeny/taxonomy has not been established. For 465 Ascomycota and Basidiomycota genomes, we predicted CAZyme-secretomes, using a new peptide-based annotation method, Conserved-Unique-Peptide-Patterns, enabling functional prediction directly from sequence. We categorized each enzyme according to CAZy-family and predicted molecular function, hereby obtaining a list of “EC-Function;CAZy-Family” observations. These “Function;Family”-based secretome profiles were compared, using a Yule-dissimilarity scoring algorithm, giving equal consideration to the presence and absence of individual observations. Assessment of “Function;Family” enzyme profile relatedness (EPR) across 465 genomes partitioned Ascomycota from Basidiomycota placing *Aspergillus* and *Penicillium* among the Ascomycota. Analogously, we calculated CAZyme “Function;Family” profile-similarities among 95 *Aspergillus* and *Penicillium* species to form an alignment-free, EPR-based dendrogram. This revealed a stunning congruence between EPR categorization and phylogenetic/taxonomic grouping of the Aspergilli and Penicillia. Our analysis suggests EPR grouping of fungi to be defined both by “shared presence“ and “shared absence” of CAZyme “Function;Family” observations. This finding indicates that CAZymes-secretome evolution is an integral part of fungal speciation, supporting integration of cladogenesis and anagenesis.

## Introduction

Classification of carbohydrate-active enzymes (CAZymes) has been investigated intensively during the last 25 years, and today the CAZymes are classified into several hundred different enzyme protein families^1^. The different types of CAZymes are divided into families based on their protein sequence similarities and their three-dimensional folding structure characteristics^1,2^. However, a CAZyme family often harbors proteins from a broad taxonomical span covering different taxonomical classes and often even different kingdoms. It has turned out that the protein sequence-based family classification to some extent matches the enzyme’s molecular function described by a specific EC number characteristic. However, many CAZyme families contain multiple types of molecular enzyme functions, i.e. the reactions the enzymes catalyze, denoted by different EC numbers^2^, and some of the larger CAZyme families have been subdivided into subfamilies by multiple alignment^3–5^. Recently, a new alignment-free clustering approach, involving the identification of Conserved Unique Peptide Patterns (CUPP), specifically assessing shared octamer peptide signatures, has been used to subdivide all CAZyme families into functionally relevant groups of proteins^6^. Since different types of carbohydrate structures, down to differences in linkage configuration, each requires specific types of unique, highly specific CAZymes for their enzymatic modification^1^ such a functional subdivision can ease the derivation of an association between fungal enzyme proteins and the specific carbohydrate carbon sources of the fungus.

It is an inherent characteristic of the heterotrophic fungal lifestyle (except for e.g. the very specialized biotrophs on animal-derived substrates) to have a broad arsenal of carbohydrate-active enzymes with functions for efficiently degrading the available biomass in their habitat. These biomass substrates are often composed of a mixture of different plant cell wall polysaccharides, primarily cellulose, hemicellulose, and pectin as well as lignin^7^. As substrate metabolism is a prerequisite for organismal fitness in growth and reproduction, the portfolio of metabolic enzymes is an essential feature in the evolutionary speciation process. Despite advances in prediction of CAZy family annotations during whole genome analyses, the current approaches do not capture the evolutionarily most important features of the enzyme profiles, namely the link between an enzyme’s protein family relation and the actual enzyme function (EC number).

Taxonomy of fungi is based upon extensive morphological and growth-related studies conducted by trained mycologists to identify exact phenotypic characteristics^7^. In some genera, notably in *Penicillium* and *Aspergillus*, specific secondary metabolite characteristics have also been included^8–11^. More recently, advances in molecular genetics have facilitated use of techniques based on DNA barcode relationships and full genome comparisons^12^. Although a few fungal species have been described to have 16,000 or more genes, and to encode for around 400 CAZymes^13^, a genome of a potent plant biomass-degrading fungus typically has a genome harboring 10,000–13,000 genes of which only 200–300 or less encode for CAZymes^7,14,15^. The types of secreted enzymes have been included in descriptions of certain fungal species^16,17^ and CAZyme gene content within the black *Aspergillus* section *Nigri* was recently included in a study of inter- and intra-species variation of these fungi^15^. A section is a formal taxonomical rank, which is an additional level between species and genus, to cope with the large variability found within the larger genera such as *Aspergillus* and *Penicillium*.

It is tempting to infer that the portfolio of secreted CAZymes is important for competitiveness, growth, and reproduction of fungi, and that they are optimized for different habitats. However, secreted CAZymes have rarely been used directly in relation to fungal taxonomy^18^, and whether a universal relationship exists between the genome-encoded profile of CAZYmes and fungal taxonomy (and phylogeny) is unclear^19–21^. It remains a challenge to predict, which genes are indeed expressed and secreted; this may differ even between strains of the same species. On the other hand, experimental secretome assessment has limitations in its ability to detect all enzyme proteins. Such experimental limitations might be overcome through genome-based prediction of the secretome.

We hypothesized that there exists a fitness-driven connection between fungal taxonomy and molecular function of the CAZymes secreted by fungi to accomplish their specialized carbon-utilization. In genome annotation, the predicted molecular function (i.e. reaction catalyzed designated as an EC number) and the CAZy family assignment was considered as one inseparable measure. For this reason we defined a combined “Function;Family“ observation to be used to assess the validity of the hypothesis. This means that two proteins with the same function, assigned to two different protein families count as two different observations (example: 3.2.1.4;GH5 is different from 3.2.1.4;GH7).

We used available genomes of fungi to create maps of fungal “Function;Family” annotated CAZyme profiles and compared the organization of these enzyme profiles to the taxonomy of the fungi. The comparative analysis of the fungal enzyme profiles was done using an algorithm that utilizes Yule distances, applying equal weight to concordantly present and concordantly absent enzyme observations. Each of the enzymes and their respective function were predicted using CUPP sequence analysis^6^. With the CUPP method at hand, the peptide signatures underlying the “conserved unique peptide patterns” of each group can be used as a prediction tool to annotate the CAZyme profiles of genomes. A match between a protein sequence obtained from the genome and the peptide signature of a CUPP group can be used to infer the molecular enzyme function, i.e. EC number, from a characterized CAZyme, belonging to the particular CUPP group. In this way, the CUPP approach can relate function to enzymes that have been associated to each other via their peptide signatures^6^. The recently developed CUPP approach has been validated on the complete set of CAZy families and demonstrated high precision, sensitivity and speed when applied for genome annotation^6^.

Using genome-based enzyme protein predictions, we here compare the “Function;Family” annotated CAZyme profiles of Ascomycota and Basidiomycota, and conduct a deep analysis of the CAZyme profiles within the *Aspergillus* and *Penicillium* genera. We report a remarkable agreement between the “Function;Family”-annotated enzyme profile relatedness (EPR)-based dendrogram and the organismal taxonomy and phylogeny of the genera *Aspergillus* and *Penicillium*. We establish that the congruence between the fungal taxonomy and the enzymatic profiles, i.e. the CAZyme secretome profiles, is based on both the enzymes that fungi in a given section commonly lack, and those they commonly share. Our analyses provide a new *in silico* predicted enzyme profile-based approach to gain insight into habitat specialization and fungal evolution. Furthermore, the findings may be of significance for identifying species/strains with specific enzymatic potential and for function-targeted enzyme discovery e.g. for improved biomass conversion.

## Results

### Connection between fungal enzyme profile relatedness and phylum taxonomy

**Figure 1:**
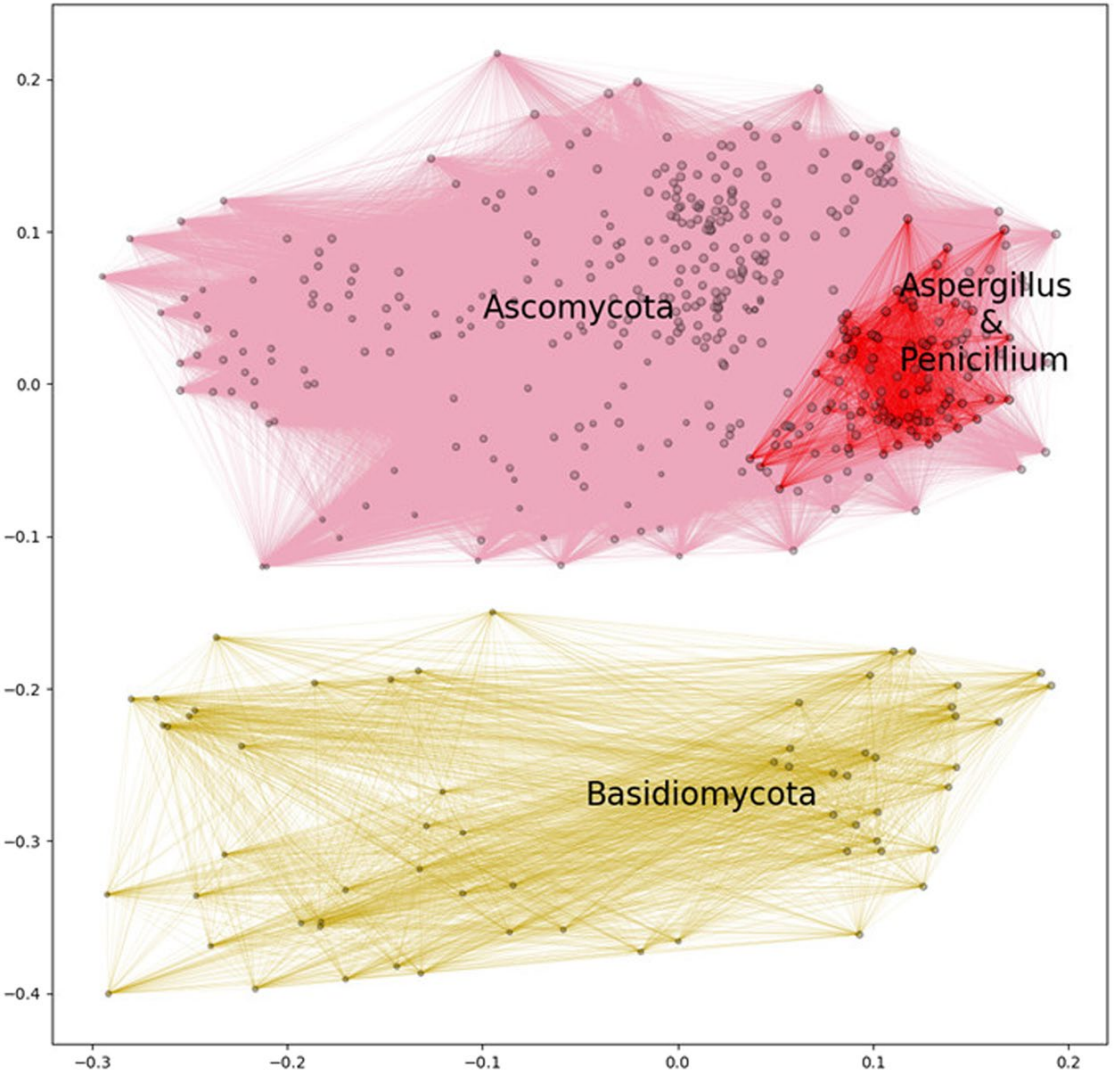
Map of selected fungi based on their predicted secreted CAZyme inventory presented as a multidimensional scaling plot. Similarity mapping of secreted “Function;Family” annotated carbohydrate active enzymes from 465 representative genomes of species of Dikarya visualized in two-dimensional space. In total 295 different enzyme “Function;Family” observations were identified. The relative sizes of the dots represent the number of different enzyme “Function;Family” observations, ranging from 40 to 144, in each genome analyzed. The distances were calculated using Yule distances based on *in silico* annotated carbohydrate active enzyme protein families combined with *in silico* prediction of enzyme function, represented by their respective EC number (if available). The clusters that represent members of the Ascomycota and Basidiomycota phyla, respectively were defined by hierarchical clustering of the calculated distances among the genomes using a flat clustering threshold of 0.7. For illustrative purposes, all species in each of these two phylum clusters are connected with pink and yellow lines, respectively. The cluster defining *Aspergillus* and *Penicillium*, containing 95 species, was based on a threshold of 0.3. All the *Aspergillus* and *Penicillium* species are connected with red lines. The coordinates were obtained by conducting 50,000 different initiations and shown as the map with the smallest final stress.

After genome filtering and prediction of secreted proteins, the secretomes of 465 Dikarya fungi (Ascomycota and Basidiomycota) were obtained. From the predicted secretomes, the CUPP method was used to annotate each protein with CAZyme family and corresponding function (EC number), and subsequently create the “Function;Family” CAZyme profile of all the secreted carbohydrate-active enzymes for each species. These profiles were arranged in a binary observation matrix with the rows outlining the fungal species and each column representing a particular “Function;Family” observation (presence or absence). From this observation matrix, a distance matrix was constructed using the Yule dissimilarity score to determine the distances between the individual fungal species based on their enzyme profile similarity, allowing assessment of enzyme profile relatedness (EPR). The species were visualized in a two-dimensional space based on the calculated distance by multidimensional scaling (MDS) (Fig. 1).

From the assessment of about 50,000 secreted CAZymes a total of 295 different “Function;Family” observations were found. The span of different enzyme “Function;Family” observations found in a single genome ranged from 40 in opportunistic human pathogenic *Trichosporon* spp. to 144 in the plant pathogenic *Diaporthe* spp. This analysis showed that enzyme profile relatedness separated the individual species of Ascomycota and Basidiomycota into their respective phyla by forming two separate and distinct clusters (Fig. 1). Furthermore, within the Ascomycota cluster, the multidimensional scaling analysis placed all species belonging to *Aspergillus* and *Penicillium* together in a compact sub-cluster, i.e. where the species of these two genera are adjacent to one another. This analysis suggests a first connection between the CAZyme secretome profile and the fungal taxonomy.

### CAZyme profiles in relation to taxonomy of *Aspergillus* and *Penicillium*

**Figure 2:**
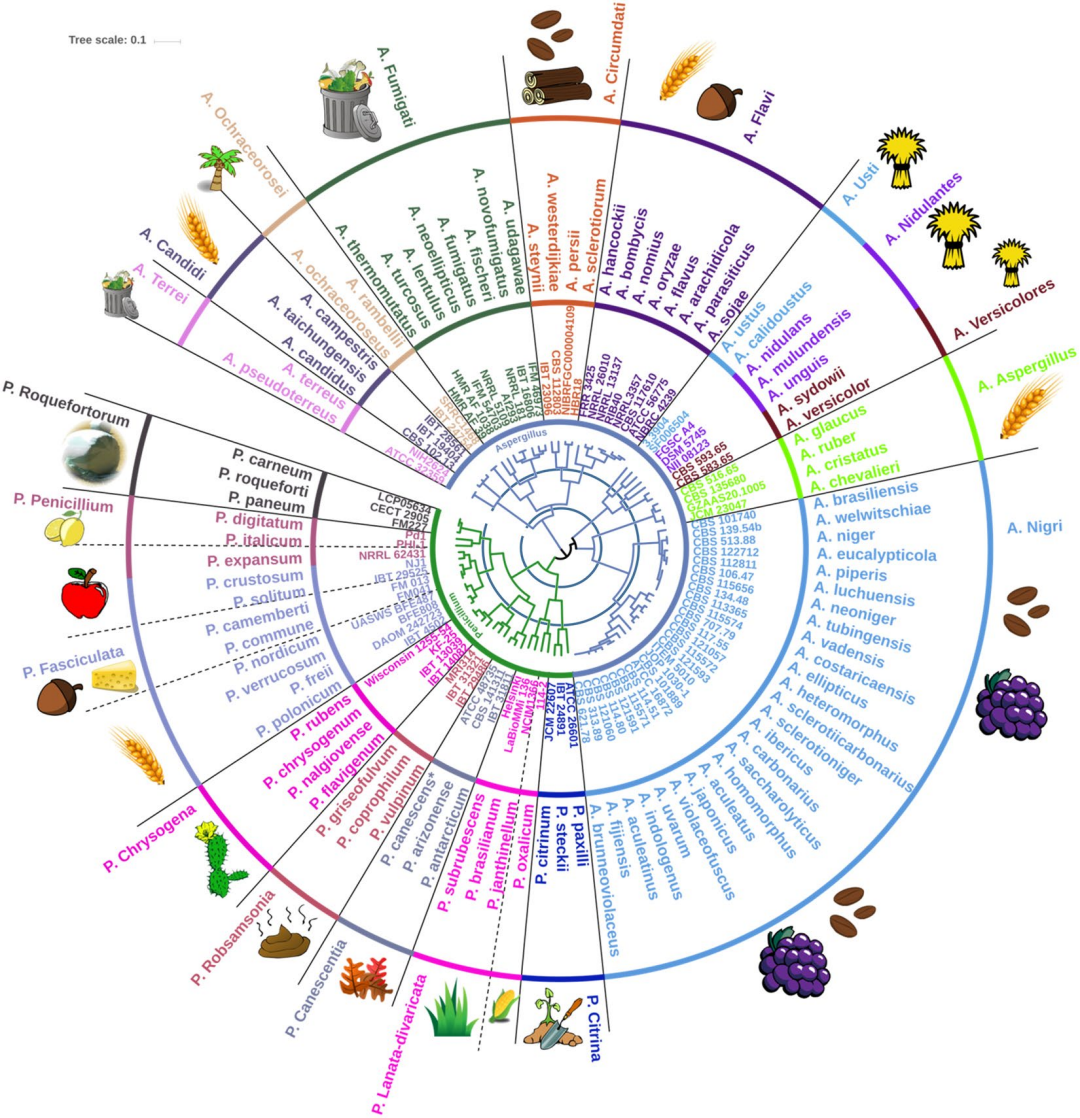
Circular dendrogram representing the secreted carbohydrate active enzyme profile relatedness, EPR, of *Aspergillus* and *Penicillium* presented with one representative genome of each of the fungal species. The distances are based on binary absence or presence assessment of “Function;Family” observation matches of the *in silico* predicted CAZyme secretomes from the genomes using Yule dissimilarity. The blue rings concentrically dividing the EPR-based dendrogram in the middle indicate the scale and have a spacing of 0.15 (innermost) and 0.3 (outermost). Circulating the dendrogram, the labels are associated to the individual genomes, as genus, strain or isolate number, species, and section, respectively. A dashed line indicates sections having members with diverse habitats or an adjacent section whose members share the same habitat. The stylized images in the outermost area indicate the primary natural habitat (or ecological specialization) of the fungal species: Clockwise description of images as they first appear, starting from section *A. Terrei*: Compost, dry Cereal, Tropical plants, Coffee, Wood, Nuts, Hay, Grapes, Plant soil, Maize, Grass, Fallen leaves, Dung, Desert plants, Cheese, Apple, Citrus and Silage. A dashed line indicates a section having more than one primary habitat. The asterisk on *P. canescens* indicates a revision of incorrect *P. capsulatum* species identification (see Supplementary Material, Fig. S2).

The two large and complex genera, *Aspergillus* and *Penicillium*, were selected to further test the hypothesis, that EPR analyses, by enzyme “Function;Family” observations would give a grouping congruent with lower taxonomic classification levels, i.e. genus, section and species. In the same way as described above for Dikarya, we took as a starting point the genome-predicted CAZyme secretomes (about 10,000 proteins in total) for *Aspergillus* and *Penicillium*, and outlined the enzyme “Function;Family” observations. Then, we employed Yule distances to assess whether the grouping of such genome-predicted enzyme observations, i.e. an enzyme profile relatedness comparison, would create a map that corresponds to fungal taxonomy and phylogeny. The strains belonging to the same species had very little, if any distance between them, analogously, species of the same section were also placed closely together (Supplementary Material, Fig. S1). Based on the enzyme profile relatedness observations, a map was constructed as a circular dendrogram and combined with the taxonomy of the 95 representative species of *Penicillium* and *Aspergillus* (Fig. 2), with few taxonomical corrections (Supplementary Material, Table S1a–e).

To obtain groupings of species (illustrated in Fig. 2), two different cut-off values were selected at 0.15 and 0.3, respectively, from the center of the dendrogram as indicated by the two blue rings. The innermost blue ring divides the members of the genus *Penicillium* into two distinct groups, one including the sections *Citrina* and *Lanata-divaricata* and the other including the remaining *Penicillium* sections. Furthermore, the innermost blue ring divides the genus *Aspergillus* into five groups: One including the section *Nigri*; one including the section *Aspergillus*; one including the sections *Usti*, *Nidulantes* and *Versicolores*; one including the sections *Flavi*, *Circumdati*, *Fumigati* and *Ochraceorosei*; and finally one including the sections *Candidi* and *Terrei*. The second cut-off at 0.3 gave the second blue ring, which forms 19 groups. Of these, nine of the groups correspond to the fungal taxonomic sections, namely: *A*. *Aspergillus* (is short-hand for section *Aspergillus* in genus *Aspergillus* (=*A*.)), *A. Candidi, P. Canescentia, A. Circumdati, P. Fasciculata (*including *P. expansum* of section *P. Penicillium), A. Flavi, A. Ochraceorosei, A. Terrei* and *A. Versicolores*. Furthermore, *A. Fumigati, P. Lanata-divaricata*, and *A. Nigri* each divided into two adjacent groups, i.e. in each case corresponding to the same fungal taxonomic sections if combined. Hence, the enzyme profile relatedness mapping was in complete accord with the fungal taxonomy for these 12 sections. The remaining fungal sections in general also grouped according to their taxonomy, although a few discrepancies were evident. Notably, the two sections *P. Chrysogena* and *P. Robsamsonia* as well as the sections *P. Roquefortorum* and *P. Penicillium* (without *P. expansum*), respectively, were found in one group, and were thus not separated by this enzyme profile relatedness grouping. However, with a slightly altered cut-off value, they would not be divided and would thus group correctly according to taxonomic section (Fig. 2).

Interestingly, *P. expansum* is located deeply within the *Fasciculata* section, close to *P. crustosum*, instead of within the *Penicillium* section as expected according to phylogenetic assessment (Supplementary Material, Fig. S3). In addition, even though the sections *Nidulantes* and *Usti* were divided into two adjacent groups, *A. nidulans* landed in the *Usti* section rather than in the *Nidulantes* section, but these sections are taxonomically quite closely related. Hence, despite these minor discrepancies, the comparison of the CAZymes secretome grouping and fungal taxonomy of *Aspergillus* and *Penicillium* (Fig. 2) provides evidence for a stunningly high degree of consensus between the CAZymes secretome EPR of the individual fungal species and their respective taxonomic grouping.

### Elucidating the group-forming EPR observation patterns

In order to elucidate the underlying reason for the strong congruence between the EPR based grouping and the taxonomy and phylogeny of *Penicillium* and *Aspergillus* (Fig. 2), several additional assessments were performed based on analysis of the enzyme “Function;Family” observations.

Interestingly, during the analysis, it was discovered, that all analyzed fungal species of *Penicillium* and *Aspergillus* share 24 enzyme “Function;Family” observations. These enzymes included laccase (EC 1.10.3.2 of AA1), LPMOs (AA9 and AA11), several glucanases of different GH families, and a number of other glycoside hydrolases belonging to families GH16, GH17, GH18, GH43, GH72, GH76, and GH132, in addition to two pectin lyases, PL1 (EC 4.2.2.10) and PL4 (EC 4.2.2.23) (Supplementary Table S2). From this, we conclude that all species of *Penicillium* and *Aspergillus* have a core set of genes encoding primarily plant cell wall degrading enzymes.

To elucidate the diversity in observations within and between the EPR groupings (Fig. 2) a measure of the total observations, and their presence and absence in the different fungal sections was determined (Table 1). This analysis made it evident that the grouping of the fungal sections appears to be formed by the observations they have in common as well as by the observations they share the absence of (Table 1).

**Table 1:**
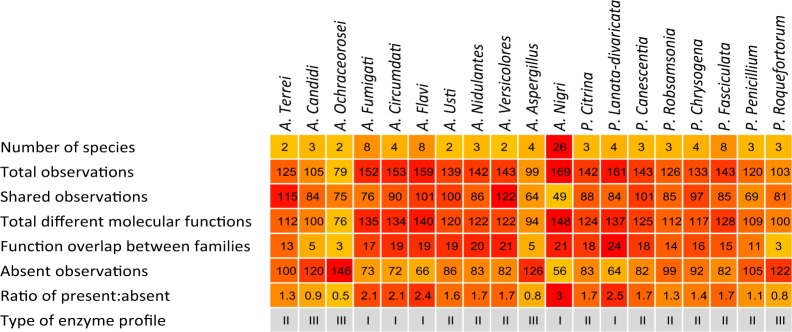
Summary of the enzyme “Function;Family” observations underlying the dendrogram in Fig. 2 organized according to the fungal sections in the dendrogram, starting from section *Terrei*.

The “Number of species” states the number of genomes included from different fungal species in each section; “Total observations” gives the number of different “Function;Family” observations in each section; “Total different functions” is the total number of different EC numbers (functions) found in the section, i.e. the number of different enzyme functions annotated from the genomes (an unknown function counts as a “function”, but does not have an EC number); “Function overlap between families” describes the number of times an EC number found in more than one CAZy family in the section; “Shared observations” describes the number of observations found within all members of a section; “Absent observations” states the number of observations that are not found in any of the members of the given section, but present in one or more of the other sections; “Ratio of present:absent” describes the proportion of the “Total observations” versus the “Absent observations”. The “present:absent ratio” obtained for the individual sections was used to assign an enzyme profile type to each section; type III, having a “present:absent ratio” below 1, indicating that members of the section are weak enzyme producers; type II having a ratio between one and two, indicating that the members of the section are medium enzyme producers; and type I, having a ratio above two, indicating the section members being strong enzyme producers.

Furthermore, based on all the enzyme “Function;Family” observations upon which the dendrogram (Fig. 2) was established, the *Aspergillus* and *Penicillium* section could be assigned to one of three types of enzyme profiles (CAZyme secretome profile type), namely type I-III, depending on their enzyme diversity capacity (Table 1). Four sections in *Aspergillus*, namely *Circumdati, Flavi, Fumigati, Nigri* and one in *Penicillium*, namely *Lanata-divaricata*, were assigned to the strong enzyme producers, Type I. These Type I EPR secretomes were grouped together primarily by the enzyme observations whose presence they share. Four sections, namely *A. Aspergillus*, *A. Candidi*, *A. Ochraceorosei* and *P. Roquefortorum*, were assigned as weak enzyme producers, enzyme profile Type III. Notably, the Type III sections were primarily grouped together by the EPR secretome observations whose absence they share. The remaining 10 sections, grouped as Type II, are categorized as moderate enzyme producers. For these sections, the groupings appeared to be a result of an almost even weighting of the enzyme observations whose presence they share versus those enzyme observations whose absence they share.

Interestingly, the sections categorized as Type I, contained both a larger number of different observations than Type III, i.e. the larger number leading to different enzyme functions (EC numbers), and also had the same function spread over a higher number of different families than the Type II and Type III enzyme profiles (“Function overlap between families”, Table 1). In contrast, the fungal sections categorized as weak enzyme producers, Type III, were found to have only a low function overlap between families meaning that the genomes of Type III members only in rare cases encode more than one family having a particular EC function. When assessing the total number of observations in the *A. Nigri* section, the diversity among the species appeared to be higher than that found in the other sections. However, the *A. Nigri* was also by far the largest section containing 28 different fungal species. With the exception of the large and quite diverse *A. Nigri* section, all members of each individual section were found to share 50% or more of their enzyme observations, indicating a high degree of homogeneity among the members of the same section. Such high enzyme profile homogeneity within the majority of the fungal sections support that members of a section share a common arsenal of CAZymes, which are likely to be related to their habitat specialization (Fig. 2).

### Elucidation of EPR-grouping of fungi in relation to habitat specialization

In general, the EPR grouping divided the fungi correctly into their taxonomical sections and this grouping simultaneously organized the fungal species according to their respective habitat specialization (Fig. 2). This finding means that the CAZyme profiles, typically based on approximately 100 CAZyme observations per fungus, can categorize fungi in accordance with their taxonomy. This indicates that fungi are indeed associated to their preferred habitat via their carbohydrate utilization ability (Fig. 2).

However, inspection of the habitat substrates, revealed that members within e.g. four of the sections, *A. Candidi*, *A. Flavi*, *A. Aspergillus*, and *P. Fasciculata*, that were otherwise spaced apart, appeared to have similar habitat specialization, namely towards dry cereal substrates (Fig. 2). To understand why these sections were divided by EPR grouping despite having similar substrate preferences, the differences in enzyme profile observations among these four sections were analyzed further (*A. Aspergillus*, *A. Candidi*, *A. Flavi* and *P. Fasciculata*, Fig. 2).

As summarized in Table 2 the most apparent differences contributing to the EPR profile discriminations are that the species in the same section either mainly share a similar set of enzyme observation or share the absence of such enzyme observations (indicated by the orange boxes, Table 2). Hence, EPR profiles distinguish the fungal sections (and species) by a combination of both the “Function;Family” observations the members of the section all have, and the observations, they share the absence of.

**Table 2:**
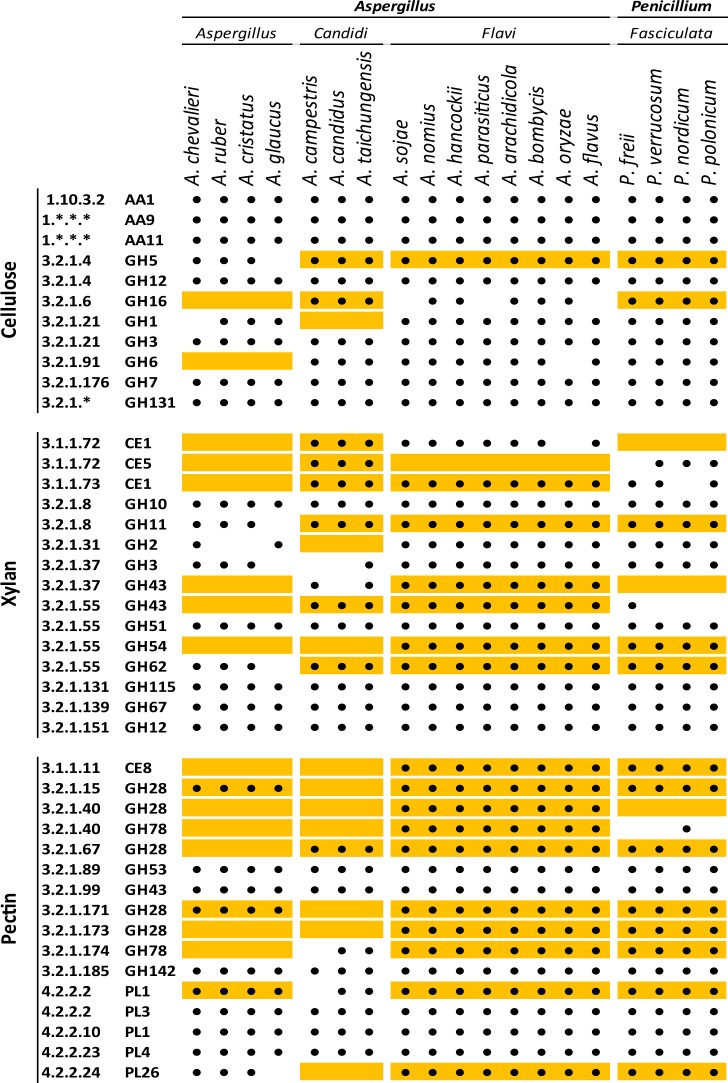
Enzyme observation overview for *A*. *Aspergillus*, *A*. *Candidi, A. Flavi* and *P*. *Fasciculata* in relation to action on the major polysaccharides cellulose, xylan, and pectin (these four fungal sections all have dry cereal as preferred habitats while being taxonomically diverse).

Orange colored cells indicate the most prominent differences between the sections, contributing to their separation with regard to EPR profile and fungal section. Dots indicate presence of an individual enzyme observation in the given fungal species; orange cells with dots indicate the presence of a particular enzyme observation (“Function;Family”) among all members of a section, except where all the included species (19 in total) have the particular observation; empty orange cells indicate enzyme observations whose absence are shared among all members of a section.

The species within all four sections have a large arsenal of enzymes active on cellulose. The species of the sections *A. Flavi* and *P. Fasciculata* essentially have similar cellulosic enzyme regime, and the differences in enzyme profiles among cellulose-active enzymes are small in the other two sections, thus the taxonomy of the fungi does not immediately appear to be explained by their capability to degrade cellulose. However, all species of section *A*. *Aspergillus* lack GH16 type endo-1,3(4)-β-glucanase (EC 3.2.1.6) and the GH6 1,4-β-cellobiosidase (non-reducing end) EC 3.2.1.91, whereas the other three sections have these two observations represented in their genomes. All species of *A. Candidi* lack GH1 β-glucosidase (3.2.1.21), but possess the GH3 family enzyme.

The greatest variation between the four sections analyzed based on their enzyme profiles, appears to be with regard to the variation in the pectin-associated enzyme observations (Table 2). More specifically, there is a general trend towards either all members of a section having a particular “Function;Family” observation or none of them having it. This finding can directly explain why the species could be organized so well in their respective sections. The two sections *A. Aspergillus* and *A. Candidi* lacked about half of the pectin-associated observations, whereas *A. Flavi* had them all. The *A. Flavi* section distanced itself by having two α-L-rhamnosidase (EC 3.2.1.40) from both family GH28 and GH78 whilst members of any of the three other sections generally lacking both (the exception being *P. nordicum* which encodes a GH78 EC 3.2.1.40 protein).

A large variation was apparent for the EPR profiles related to xylan modification. The analysis revealed a maximum variability for the enzyme observations designating acetyl xylan esterase (EC 3.1.1.72) of family CE1 and CE5 in relation to their shared presence and absence in the four sections. *A*. *Candidi* had both CE1 and CE5 (EC 3.1.1.72), whereas *A*. *Flavi* members encoded CE1 (except one of the species) but not CE5, whilst P. *Fasciculata* only encoded CE5, and finally *A*. *Aspergillus* encoded no acetyl xylan esterases at all (Table 2). A large variation among the α-arabinofuranosidase functions (EC 3.2.1.55) was also evident, and likely contributed to the discrimination. Hence, CUPP identified presence of EC 3.2.1.55 from GH51 and GH62 in all sections; but, similarly to the acetyl xylan esterase case, the EPR profiling showed maximum variability for the EC 3.2.1.55 GH43 and GH54. Thus, members of the *A*. *Flavi* section encoded both, all members of *A*. *Candidi* encoded GH43 but not GH54, and all members of *P*. *Fasciculata* had GH54, and generally not GH43 (only one member encoded for GH43), whereas *A*. *Aspergillus* encodes none of these (Table 2). Families with the highest number of different molecular functions have the potential to contribute most to the phylogenetic and phenetic differentiation. In the data set two families, GH5 and GH28, have the highest number of molecular functions.

## Discussion

The secretome of carbohydrate metabolizing enzymes is under particularly heavy evolutionary pressure as it provides the basis for the fungal growth and reproduction in Nature. Since growth-related characteristics related to the ability to metabolize different substrates is a central element in fungal biology, it is logical to infer that there must be a connection between the enzymes fungi secrete to accomplish their specialized carbon-utilization and their taxonomy and phylogeny. To our knowledge, it has not been proposed before that the fungal secretome is important for speciation. Rather, it has been the philosophy that functional characters cannot be used for phylogeny^22^, but instead reflects ecological selection and adaptation capabilities. Phylogeny these days is based on house-hold gene sequence comparison. Only in rare cases are any functional genes included for phylogenetic reconstruction^21^. Hence, a comparison of the enzymes fungi secrete in relation to fungal evolution has in general been considered futile as the plasticity of fungal evolution and enzyme secretome changes were considered too high to obtain a meaningful relation^22–24^. The current study, however, affirmed the validity of the conceptual idea of a link between CAZy secretome relatedness and fungal taxonomy, as exemplified by analysis of the two highly complex genera *Penicillium* and *Aspergillus*.

Most enzyme phylogenies are alignment-based and mainly useful for comparing relatively similar enzyme sequences with each other^3–5,25^. Here, in contrast to assessing similarities of aligned columns of amino acids in protein sequences, we introduced a broader non-alignment CAZyme comparison approach based on “Function;Family” enzyme predictions. By assigning the “Function;Family” connection and comparing the enzyme profile relatedness from fungal secretomes, we capture the feature that fungal genomes often harbor capacity to produce several different types of enzymes, belonging to different families for the same function. This feature reflects the plasticity, capacity, and diversity of the fungal genome. The comparison of presence or absence of predicted “Function;Family” observations are the foundation thus serving as building blocks of the EPR calculation. Since it was evident that the contribution of the absent observations were of significance when comparing EPR profiles (Table 2), we used Yule distances^26^ and not e.g. Jaccard similarity for the enzyme profile relatedness assessment. The use of the Yule measure for calculation of distances between the enzyme profiles of different genomes considers presence and absence equally and thus evaluates gene gain and loss with equal weight^26^. The Yule dissimilarity score^27^ is particularly useful for correlations among binary character profiles. It differs from the renowned Jaccard similarity coefficient by including zero-zero pairs in the scoring. Based on this it was obvious to choose the Yule dissimilarity score to calculate the EPR based dendrogram. In this study, the *ab initio* predicted genes containing a signal peptide are expected to be of importance. Experimental secretome identification could potentially reveal that some of the encoded CAZymes are only actively expressed through induction by certain carbon sources. However, the limitation of experimental secretome assessment may lead to undetected proteins or false positives.

The comparison of the CAZyme secretome profiles across the Ascomycota and Basidiomycota showed that the CUPP-based, “Function;Family” calculated EPR approach was robust across large phylogenetic distances. It was a striking finding that the CAZyme-based EPR map of fungi (Fig. 1) divided these two large phyla in two distinct groups. In addition, the EPR analysis also revealed the subcluster of *Aspergillus* and *Penicillium* within the Ascomycota. A direct comparison of the abundance of the 24 enzyme observations found in all *Aspergillus* and *Penicillium* (Supplementary Table S2) further corroborated the separate EPR-based clustering of species of Ascomycota and Basidiomycota shown in the MDS plot (Fig. 1). This clustering map of the Dikarya was a first indication that the genes encoding CAZymes dominate the genomic discrepancies of the fungi, and that the “Function;Family”-based annotation of CAZyme secretome profiles are directly connected to the taxonomy and phylogeny of fungi. This study thus presents the first proof of principle for the validity of EPR assessment (calculated based on “Function;Family” annotation) as a basis for secretome comparison across the fungal kingdom.

The EPR-based dendrogram of *Penicillium* and *Aspergillus* CAZyme secretomes (Fig. 2) coincided to a stunning extent with phylogenies of the two genera based on household genes (Figs. S2–S4), as they appear to group the species and sections congruently. This congruence is particularly striking considering that the CAZymes diversity is only conferred by 200–300 genes out of the total fungal genome of 10,000–13,000 genes equivalent to about 2% of the total gene pool. Many household-genes are appropriate for phylogenetic analysis. In *Penicillium* and *Aspergillus* the β-tubulin gene is one of the most commonly recommended genes for phylogenetic analysis^10,28,29^. β-tubulin was used also in this study. In addition, to make a more robust multi-gene phylogeny, the heat shock protein (HSP) and other tubulin genes were added, as they could be extracted from the genomes of the fungi considered.

Regarding *P. canescens*, there appears to be an inconsistency in the taxonomy. The two strains LiaoWQ-2011 (synonym CBS 134186) and ATCC 48735 are designated as *P. capsulatum* (section *Ramigena*) according to NCBI. In the original publication the morphology and the ITS of the two strains of *P. capsulatum* seems correctly identified^30^ when compared to the *P. capsulatum* type strain CBS 301.48. However, the ITS barcode (JX841248) listed in the study^30^ did not match the ITS found within the genomes of the two *P. capsulatum* strains, LiaoWQ-2011 (= CBS 134186) and ATCC 48735. Rather, the ATCC 48735 (used here, Fig. 2), has been coined as being *P. canescens*. The comparison revealed a close relationship between the ITS sequences found in the genomes and the ITS barcodes from the BOLD Systems database^31^ for *P. antarcticum* and *P. arizonense* of section *Canescentia*. This finding was also in agreement with the results of EPR where the strains were placed in section *Canescentia* along with *P. arizonense* and *P. antarcticum*.

Despite the essential role in fungal evolution of secreted proteins that metabolize and degrade substrates to accessible molecules, these functional proteins have rarely, if ever, been included in classification and never in cladification. By using the “Function;Family” observations, we capture a key feature of the heterotrophic fungi, namely that they have developed several different types of enzymes to accomplish the same function, and find that the enzyme profiles consist of a selection of specific enzyme functions of prime importance for growth and reproduction of the organism on a specific substrate. The extent and type of CAZyme profile differences varied significantly between different categories of enzymes (Table 2).

Many of the enzyme functions needed for degrading cellulose were present throughout many sections and therefore would not be defining for the grouping. In contrast, for xylan, a diversity was evident, but a nuanced assessment of “Function;Family” differences required extracting what was defining for the grouping: If molecular function was only considered for e.g. EC 3.2.1.55 without considering the CAZy-family delineation, the discrimination between the different fungal profiles would not have been evident. Hence, for the individual observations the tying of CAZyme family to the molecular function enabled a more nuanced discrimination among the enzyme profiles, displaying both a) differences in evolutionary outcomes for solving the same problem (e.g. hydrolytic cleavage of arabinofuranosyl bonds in arabinoxylans) and b) the detailed differentiated “Function;Family” CAZymes profiles matching the fungal taxonomy and phylogeny. Hence, what is captured by the “Function;Family” observations, i.e. the families of CAZymes that are available for which type of functions, is an integrated part of evolution of species and therefore also of their taxonomic position.

*Aspergillus* and *Penicillium* have been intensively studied with regard to their speciation, morphology and physiological specializations^10,29,31–33^. They inhabit and metabolize, respectively, a vast range of habitats and substrates and are therefore a highly suitable, albeit challenging, case study for comparison of taxonomy to the calculated enzyme profile relatedness here. Some species, for example those in *P. Robsamsonia*, are adapted to animal dung^33^, where most degradable carbohydrate substrates have already been metabolized in the animal gut, leaving very recalcitrant polysaccharides and lignins unmetabolized. Therefore, unique enzymes are needed to be strongly associated to such substrates. In *Penicillium* section *Fasciculata* there is a group of species adapted to dry cereals (*P. verrucosum, P. freii, P. polonicum*) and some growing predominantly on substrates rich in lipid and protein such as cheese (*P. camemberti* and *P. commune*)^31^. Stress-selected fungi such as the species in section *A. Aspergillus*^32,34–36^, which grow mostly on substrates with very low water activity, had fewer secreted enzymes than species from other *Aspergillus* sections that are primarily competition-selected. Species in other *Aspergillus* sections grow in environments more competitive and produce more secondary metabolites and a much larger number of secreted enzymes^34^.

EPR analysis of observations found in each of the EPR groupings suggests that EPR groupings are sustained both by the observations shared in the group and by the observations, whose absence they share. Interestingly, the ratio between such positive and negative observations varies significantly between sections. Furthermore, the study also analyzed which observations underpin why species of *Aspergillus* and *Penicillium* with the same substrate affinity (e.g. dry cereal biomass) are found in different sections and here both the present and absent observations are shown to be of importance. By constructing an EPR-based dendrogram based on “Function;Family” annotation of CAZyme secretomes a striking equivalence between the relatedness of groupings obtained through EPR analysis and the taxonomic grouping of fungal species was found. This finding supports the inference that through evolution, characteristic enzyme profiles of the fungal carbohydrate-active secretomes have been developed for each of the sections of these two genera. This finding implies that the evolutionary development of the metabolic enzyme secretome is an integral part of the evolutionary speciation process^8^. This study shows that secreted enzymes are strong classificatory and cladificatory markers and could be used more extensively in taxonomy and phylogeny. The EPR approach may also enable targeted enzyme discovery.

## Methods

### Genome protein prediction and annotation

Full genome sequences for about 2,000 fungi were downloaded from the National Center for Biotechnological Information (NCBI) in September 2018. Representative genomes covering all available genomes of Basidiomycota and Ascomycota, with the exception of species belonging to the taxonomical class Saccharomycetes were included. For quality assurance, only assemblies belonging to a genus with at least four assemblies were included and only if their taxonomical identifier could be confirmed by phylogenetic assessment, as described below. The taxonomic data were obtained from the associated NCBI assembly reports of the individual genomes and in a few cases manually revised (Supplementary Material, Table S1a–e). For Ascomycota, the encoding genes and corresponding proteins were predicted using Augustus 2.5 with model *Aspergillus oryzae*^37^. For all Basidiomycota genomes coding genes were found using the *Ustilago* model. Predicted proteins were analyzed by SignalP 4.1^38^, Phobius^39^ and Wolf PSORT^40^, and were annotated as secreted when predicted by at least two of the three tools. Proteins predicted to be secreted were annotated using CUPP^6^ for carbohydrate-active protein domains where a positive hit counted as an observation. Only presence or absence of an observation was considered, and multiple occurrences of the same observation (within one genome) were considered redundant and only counted as one. An observation was defined as a string combining the predicted CAZy protein family name with the predicted EC number of target protein “protein family:molecular function” e.g. GH1:3.2.1.37. A function of an enzyme was defined as the EC number description. In case one protein was predicted to have an indecisive functional annotation between two molecular functions, both were counted as a half (a score above one count as present whereas a score below one counts as absent). For the Dikarya processing, observations only found in a single genome were ignored to reduce the potential influence of low quality genomes. The observations were recorded in an “m” times “n” observation matrix where “m” is the number of genomes analyzed and “n” is the total number of different observed function-family combinations. From the observation matrix, an “m” times “m” distance matrix was obtained using the Yule distance metric from the scipy.spatial.distance Python package version 1.3.0. The Yule distance equation:

$$Yul{e}_{dissimilarity\_score}=\frac{2\cdot {C}_{TF}\cdot {C}_{FT}}{{C}_{TT}\cdot {C}_{FF}+{C}_{TF}\cdot {C}_{FT}}$$

where ${C}_{TT}$ is the number of different observation two entities share the presence of, ${C}_{FF}$ is the number of different observation two entities share the absence of, and ${C}_{FT}$ or ${C}_{TF}$ are the observations found in one entity but not in the other. The distance matrix was visualized in a dendrogram by complete linkage and in a MDS plot using the scipy.cluster.hierarchy.linkage, scipy.cluster.hierarchy.dendrogram and sklearn.manifold.MDS function, respectively^41^. The clusters were obtained through flat clustering with a threshold of 0.3 or 0.7 for genera and phyla, respectively, using the criterion “distance”, further described in the function scipy.cluster.hierarchy.fcluster.

### Filtration of genomes

Genome entities listed as partial in the NCBI statistic report were disregarded along with genomes of poor quality defined according to the following criteria. For establishment of the phylogeny of the Ascomycota and Basidiomycota four barcodes were assessed (HSP88 (XP_001392647), HSP90 (XP_001393974.1), α-1-tubulin (XP_001388988.1) and β-tubulin (XP_001392436.1)). These proteins were identified by using the reference sequences of *A. niger* CBS 513.88 as a query for BLAST against the protein lists of the individual genomes for identification of orthologous proteins. Is case an orthologous protein could not be identified, the fungi were not further considered. Each kind of orthologous protein was aligned separately for subsequent concatenation using MEGA7^42^ for multiple alignments with MUSCLE scoring^43^. The concatenation alignment was used for construction of a traditional Neighbors-end joining^44^ phylogenetic tree using pairwise deletion and 100 bootstrap iterations. This was done to establish a deeper understanding of the taxonomic relationship between the species and to identify irregularities in the taxonomic identification listed in NCBI. Entities not placed consistently near the taxonomically related species i.e. not having at least three species from the same genus next by, were not considered for further analysis. A combined phylogenetic tree is available in Supplementary Material, Fig. S4. Furthermore, genomes having less than 40 different “Function;Family” observations were not included in further analysis to secure enough information in each entity for proper separation.

For each of the genomes of *Aspergillus* and *Penicillium*, 14 proteins (related to Tubulin or Heat Shock Proteins) were attempted to be identified by BLAST as described above. This was done to validate the provided taxonomical identification listed in NCBI to supply bases for removal or renaming of certain fungi for further analysis. Fourteen orthologues proteins were used from each *Aspergillus* and *Penicillium* species to obtain required resolution to observe differences down to species level. In total six Tubulin proteins (α-1-Tubulin (XP_001388988.1), α-2-Tubulin (XP_001396857.1), β-1 (XP_001392436.1), γ-Tubulin (XP_001392761.2), Tubulin-specific chaperone C (XP_001397556.1) and Tubulin-specific chaperone D (XP_001398704.2)) and eight Heat Shock Proteins were included (HSP60 (XP_001395564.1), HSP70-1 (XP_025459513.1), HSP70-2 (XP_025448922.1), HSP88 (XP_001392647), HSP90 (XP_001393974.1), HSP98-1 (XP_001392464.1), HSP98-1 (XP_001389736.1) and HSP-STI1 (XP_001395168.2)). The resulting phylogenetic tree can be seen the Supplementary Fig. S3. Species that were alone within their own section were not considered for further analysis; when multiple strains of the same species were available, only the newest version was considered (a dendrogram containing all strains can be seen in Fig. S1). The *Aspergillus* strain Z5 has been assigned to the species *A. sydowii*^45^; *Penicillium* strain HKF2 is listed as *P. chrysogenum*^46^; Three strains (NCPC10086, P2niaD18, IB 08/921) listed as *P. chrysogenum* have been altered to *P. rubens*^47,48^.

As a result of the filtering procedure several different species have been corrected based on Supplementary Materials Figs. S2–S4, Table S1a–e and Table S2.

## Supplementary information

Supplementary information.
