# Supplementary material for: Fungal secretome profile categorization of CAZymes by function and family corresponds to fungal phylogeny and taxonomy: Example Aspergillus and Penicillium
Source: Sci Rep. 2020 Mar 20;10:5158. doi: 10.1038/s41598-020-61907-1 (PMC7083838; doi:10.1038/s41598-020-61907-1)
Supplement: Supplementary file 1 — Supplementary information. [file 41598_2020_61907_MOESM1_ESM.pdf]

Supplementary material - Fungal secretome profile categorization of CAZymes by function and family corresponds to the taxonomy and phylogeny of fungi: Example *Aspergillus* and *Penicillium*

Kristian Barrett[1], Kristian Jensen[2], Anne S. Meyer[1], Jens C. Frisvad[1‡*] and Lene Lange[3‡]

[1]Department for Biotechnology and Biomedicine, Building 221, Technical University of Denmark, DK-2800 Lyngby, Denmark

[2]The Novo Nordisk Foundation Center for Biosustainability, Building 220, Technical University of Denmark, DK-2800 Lyngby, Denmark

[3]LLa Bioeconomy, Research & Advisory, Karensgade 5, DK-2500 Valby, Denmark.

[*]Corresponding author, mail jcf@bio.dtu.dk.

[‡]Shared senior authorship.

Keywords: Enzyme Profile Relatedness; Function in Relation to Protein Family; Conserved Unique Peptide Patterns CUPP; Functional Genome Comparison; CAZymes; Fungal secretome evolution

Table S1 (a-e) Strains belonging to the genera *Aspergillus* or *Penicillium* along with their reported taxonomic section, the species used in the current study, the species supplied by NCBI, the strain or isolate number, the NCBI accession number and a "Note" column. An "X" in the Note column indicates a representative strain of the given species whereas an empty field indicates a strain which was not selected as representative strain for the species in the current work. The genome assemblies resulting from re-sequencing, is treated as one and the excluded genome assembly is denoted as "DUPLET". Sections having only a single genome assembly available is not included in the current work, denoted "ALONE". The star on the actual species name and the note "ODD" indicates that the taxonomy (identity) of the strain could not be supported through assessment of the alignment based phylogenetic tree of 14 orthologous proteins. A bold species name indicates a correction of the species name used in the current work.

Table S1a

Genus	Section	Actual species	Species of NCBI	Strain/Isolate	Accession NCBI	Note
Aspergillus	*Aspergillus*	*A. chevalieri*	*A. chevalieri*	JCM 23047	GCA_001599875.1	X
		A. cristatus	*A. cristatus*	GZAAS20.1005	GCA_001717485.1	X
		A. cristatus	*A. cristatus*	YKY807	GCA_001693355.1	
		A. glaucus	*A. glaucus*	CBS 516.65	GCA_001890805.1	X
		A. ruber	*A. ruber*	CBS 135680	GCA_000600275.1	X
	Candidi	*A. campestris*	*A. campestris*	IBT 28561	GCA_002847485.1	X
		A. candidus	*A. candidus*	CBS 102.13	GCA_002847045.1	X
		A. taichungensis	*A. taichungensis*	IBT 19404	GCA_002850765.1	X
	Circumdati	*A. persii*	*A. persii*	NIBRFGC000004109	GCA_002215965.1	X
		A. sclerotiorum	*A. sclerotiorum*	HBR18	GCA_000530345.1	X
		A. steynii	*A. steynii*	IBT 23096	GCA_002849105.1	X
		A. westerdijkiae	*A. westerdijkiae*	CBS 112803	GCA_001307345.1	X
	Clavati	*A. clavatus*	*A. clavatus*	NRRL 1	GCA_000002715.1	ALONE
	Cremei	*A. wentii*	*A. wentii*	DTO 134E9	GCA_001890725.1	ALONE
	Flavi	*A. arachidicola*	*A. arachidicola*	CBS 117610	GCA_002749805.1	X
		A. arachidicola	***A. oryzae***	ATCC 12892	GCA_002894705.1	
		A. bombycis	*A. bombycis*	NRRL 26010	GCA_001792695.1	X
		A. flavus	*A. flavus*	NRRL 21882	GCA_002217635.1	DUPLET
		A. flavus	*A. flavus*	NRRL3357	GCA_000006275.2	X
		A. flavus	*A. flavus*	AF70	GCA_000952835.1	
		A. flavus	*A. flavus*	79-2	GCA_001576635.1	
		A. flavus	*A. flavus*	61-4	GCA_001576645.1	
		A. flavus	*A. flavus*	40-5	GCA_001576655.1	
		A. flavus	*A. flavus*	26-3	GCA_001576715.1	
		A. flavus	*A. flavus*	78-6	GCA_001576725.1	
		A. flavus	*A. flavus*	72-5	GCA_001576735.1	
		A. flavus	*A. flavus*	206-4	GCA_001576745.1	
		A. flavus	*A. flavus*	54-2	GCA_001576795.1	
		A. flavus	*A. flavus*	3-2	GCA_001695535.1	
		A. flavus	*A. flavus*	JAU2	GCA_002217615.1	
		A. flavus	*A. flavus*	NRRL 21882	GCA_002443195.2	
		A. flavus	*A. flavus*	NRRL 30797	GCA_002443215.2	
		A. flavus	*A. flavus*	NRRL 118543	GCA_002456175.2	
		A. flavus	*A. flavus*	WRRL1519	GCA_002864195.1	
		A. hancockii	*A. hancockii*	FRR 3425	GCA_001696595.1	X
		A. nomius	*A. nomius*	NRRL 13137	GCA_001204775.1	X
		A. nomius	*A. nomius*	HBR9	GCA_000531055.1	
		A. oryzae	*A. oryzae*	RIB40	GCA_000184455.3	DUPLET
		A. oryzae	*A. oryzae*	RIB40	GCA_000965245.1	X
		A. oryzae	*A. oryzae*	3.042	GCA_000269785.2	
		A. oryzae	*A. oryzae*	AS 3.951	GCA_000278405.1	
		A. oryzae	*A. oryzae*	AS 3.863	GCA_000278425.1	
		A. oryzae	*A. oryzae*	RIB326	GCA_000320905.1	
		A. oryzae	*A. oryzae*	100-8	GCA_000691885.1	
		A. oryzae	*A. oryzae*	BCC7051	GCA_002007945.1	
		A. oryzae	*A. oryzae*	SRCM101975	GCA_002214955.1	
		A. oryzae	*A. oryzae*	SRCM101989	GCA_002214965.1	
		A. parasiticus	*A. parasiticus*	ATCC 56775	GCA_000731695.1	X
		A. parasiticus	*A. parasiticus*	SU-1	GCA_000956085.1	
		*A. parasiticus**	*A. parasiticus*	68-5	GCA_001576805.1	ODD
		A. sojae	*A. sojae*	NBRC 4239	GCA_000226655.1	X

Table S1b

Genus	Section	Actual species	Species of NCBI	Strain/Isolate	Accession NCBI	Note
Aspergillus	*Fumigati*	*A. fischeri*	*A. fischeri*	NRRL 181	GCA_000149645.2	X
		A. fumigatus	*A. fumigatus*	Af293	GCA_000002655.1	X
		A. fumigatus	*A. fumigatus*	A1163	GCA_000150145.1	
		A. fumigatus	*A. fumigatus*	AF10	GCA_000225625.2	
		A. fumigatus	*A. fumigatus*	AF210	GCA_000225645.2	
		A. fumigatus	*A. fumigatus*	niveus	GCA_000731615.1	
		A. fumigatus	*A. fumigatus*	CGMCC 3309	GCA_001029325.1	
		A. fumigatus	*A. fumigatus*	JCM 10253	GCA_001599455.1	
		A. fumigatus	*A. fumigatus*	IF1SW-F4	GCA_001643655.1	
		A. fumigatus	*A. fumigatus*	ISSFT-021	GCA_001643665.1	
		A. fumigatus	*A. fumigatus*	LMB-35Aa	GCA_001715275.2	
		A. fumigatus	*A. fumigatus*	HMR AF 270	GCA_002234955.1	
		A. fumigatus	*A. fumigatus*	HMR AF 706	GCA_002234985.1	
		*A. fumigatus**	*A. fumigatus*	SPS-2	GCA_003069565.1	ODD
		A. lentulus	*A. lentulus*	IFM 54703	GCA_001445615.1	X
		A. neoellipticus	*A. neoellipticus*	NRRL 5109	GCA_003116565.1	X
		A. novofumigatus	*A. novofumigatus*	IBT 16806	GCA_002847465.1	X
		A. thermomutatus	*A. thermomutatus*	HMR AF 39	GCA_002237265.1	X
		A. turcosus	*A. turcosus*	HMR AF 1038	GCA_002234975.1	X
		A. turcosus	*A. turcosus*	HMR AF 23	GCA_002234965.1	
		A. udagawae	*A. udagawae*	IFM 46973	GCA_001078395.1	X
	Nidulantes	*A. mulundensis*	*A. mulundensis*	DSM 5745	GCA_003369625.1	X
		A. nidulans	*A. nidulans*	FGSC A4	GCA_000011425.1	DUPLET
		A. nidulans	*A. nidulans*	FGSC A4	GCA_000149205.2	X
		A. unguis	*A. unguis*	NII 08123	GCA_003324175.1	X
	Ochraceorosei	*A. ochraceoroseus*	*A. ochraceoroseus*	IBT 24754	GCA_002846915.2	X
		A. ochraceoroseus	*A. ochraceoroseus*	SRRC1432	GCA_000986665.1	
		A. rambellii	*A. rambellii*	SRRC1468	GCA_000986645.1	X
	Terrei	*A. pseudoterreus*	*A. pseudoterreus*	ATCC 32359	GCA_002927005.1	X
		A. terreus	*A. terreus*	NIH2624	GCA_000149615.1	X
		A. terreus	*A. terreus*	45A	GCA_001630395.1	
		A. terreus	*A. terreus*	IMV 01167	GCA_001931935.1	ODD
		A. terreus	*A. terreus*	w25	GCA_002749855.1	
		*A. terreus**	*A. terreus*	T3_Kankrej	GCA_002930435.1	ODD
	Usti	*A. calidoustus*	*A. calidoustus*	SF006504	GCA_001511075.1	X
		A. ustus	*A. ustus*	3.3904	GCA_000812125.1	X
	Versicolores	*A. sydowii*	*A. sydowii*	CBS 593.65	GCA_001890705.1	X
		A. sydowii	***A. spp.***	Z5	GCA_001044295.1	
		A. versicolor	*A. versicolor*	CBS 583.65	GCA_001890125.1	X
		A. versicolor	*A. versicolor*	MA 6037	GCA_003138035.1	
		*A. versicolor**	*A. versicolor**	MA 6041	GCA_003138005.1	ODD

Table S1c

**Genus**	**Section**	**Actual species**	**Species of NCBI**	**Strain/Isolate**	**Accession NCBI**	**Note**
Aspergillus	*Nigri*	*A. aculeatinus*	*A. aculeatinus*	CBS 121060	GCA_003184765.1	X
		A. aculeatus	*A. aculeatus*	ATCC 16872	GCA_001890905.1	X
		A. brasiliensis	*A. brasiliensis*	CBS 101740	GCA_001889945.1	X
		A. brunneoviolaceus	*A. brunneoviolaceus*	CBS 621.78	GCA_003184695.1	X
		A. carbonarius	*A. carbonarius*	ITEM 5010	GCA_001990825.1	X
		A. costaricaensis	*A. costaricaensis*	CBS 115574	GCA_003184835.1	X
		A. ellipticus	*A. ellipticus*	CBS 707.79	GCA_003184645.1	X
		A. eucalypticola	*A. eucalypticola*	CBS 122712	GCA_003184535.1	X
		A. fijiensis	*A. fijiensis*	CBS 313.89	GCA_003184825.1	X
		A. heteromorphus	*A. heteromorphus*	CBS 117.55	GCA_003184545.1	X
		A. homomorphus	*A. homomorphus*	CBS 101889	GCA_003184865.1	X
		A. ibericus	*A. ibericus*	CBS 121593	GCA_003184845.1	X
		A. indologenus	*A. indologenus*	CBS 114.80	GCA_003184685.1	X
		A. japonicus	*A. japonicus*	CBS 114.51	GCA_003184785.1	X
		A. violaceofuscus	*A. violaceofuscus*	CBS 115571	GCA_003184705.1	X
		A. luchuensis	*A. luchuensis*	CBS 106.47	GCA_001890685.1	X
		A. luchuensis	***A. kawachii***	IFO4308	GCA_000239835.2	
		A. luchuensis	***A. awamori***	JCM 22320	GCA_001599415.1	
		A. luchuensis	*A. luchuensis*	RIB 2604	GCA_001602395.1	
		A. luchuensis	***A. niger***	An76	GCA_001515345.1	
		A. neoniger	*A. neoniger*	CBS 115656	GCA_003184625.1	X
		A. niger	*A. niger*	CBS 513.88	GCA_000002855.2	X
		A. welwitschiae	*A. welwitschiae*	CBS 139.54b	GCA_003344945.1	X
		A. niger	*A. niger*	ATCC 1015	GCA_000230395.2	
		A. niger	*A. niger*	SH-2	GCA_000633045.1	
		A. niger	*A. niger*	ATCC 10864	GCA_001715265.1	
		A. niger	*A. niger*	A1	GCA_001741885.1	
		A. niger	*A. niger*	H915-1	GCA_001741905.1	
		A. niger	*A. niger*	L2	GCA_001741915.1	
		A. niger	*A. niger*	JSC-093350089	GCA_001931795.1	
		A. niger	*A. niger*	FDAARGOS_311	GCA_002211485.1	
		A. niger	*A. niger*	FGSC A1279	GCA_002740505.1	
		A. niger	***A. lacticoffeatus***	CBS 101883	GCA_003184595.1	
		A. niger	***A. phoenicis***	ATCC 13157	GCA_003344505.1	
		A. niger	*A. niger*	ATCC 13496	GCA_003344705.1	
		A. niger	*A. niger*	ATCC 64974	GCA_900248155.1	
		*A. niger**	*A. niger**	COH1141	GCA_003123655.1	ODD
		A. piperis	*A. piperis*	CBS 112811	GCA_003184755.1	X
		A. saccharolyticus	*A. saccharolyticus*	JOP 1030-1	GCA_003184585.1	X
		A. sclerotiicarbonarius	*A. sclerotiicarbonarius*	CBS 121057	GCA_003184635.1	X
		A. sclerotioniger	*A. sclerotioniger*	CBS 115572	GCA_003184525.1	X
		A. tubingensis	*A. tubingensis*	CBS 134.48	GCA_001890745.1	X
		A. tubingensis	*A. tubingensis*	Atub1	GCA_900163765.1	
		A. uvarum	*A. uvarum*	CBS 121591	GCA_003184745.1	X
		A. vadensis	*A. vadensis*	CBS 113365	GCA_003184925.1	X

Table S1d

Genus	Section	Actual species	Species of NCBI	Strain/Isolate	Accession NCBI	Note
Penicillium	*Canescentia*	*P. antarcticum*	*P. antarcticum*	IBT 31811	GCA_002072345.1	X
		P. arizonense	*P. arizonense*	CBS 141311	GCA_001773325.1	X
		P. canescens*	***P. capsulatum***	ATCC 48735	GCA_000943765.1	X
		P. canescens*	***P. capsulatum***	LiaoWQ-2011	GCA_000943775.1	
		P. chrysogenum	*P. chrysogenum*	KF-25	GCA_000816005.1	X
		P. chrysogenum	***P. spp.***	HKF2	GCA_002000375.1	
		P. flavigenum	*P. flavigenum*	IBT 14082	GCA_002072365.1	X
		P. nalgiovense	*P. nalgiovense*	IBT 13039	GCA_002072425.1	X
		P. nalgiovense	*P. nalgiovense*	FM193	GCA_000577395.2	
		P. nalgiovense	*P. nalgiovense*	CF05	GCA_002916455.1	
		P. rubens	*P. rubens*	Wisconsin 1255-54	GCA_000226395.1	X
		P. rubens	***P. chrysogenum***	HKF42	GCA_002080375.1	
		P. rubens	***P. chrysogenum***	NCPC10086	GCA_000523475.1	
		P. rubens	*P. rubens*	P2niaD18	GCA_000710275.1	
		P. rubens	***P. chrysogenum***	IB 08/921	GCA_000801355.1	
		P. rubens	***P. spp.***	MA 6040	GCA_003138025.1	
	Citrina	*P. citrinum*	*P. citrinum*	JCM 22607	GCA_001950535.1	X
		P. citrinum	*P. citrinum*	DSM 1997	GCA_001399475.1	
		P. paxilli	*P. paxilli*	ATCC 26601	GCA_000347475.1	X
		P. steckii	*P. steckii*	IBT 24891	GCA_002072375.1	X
	Exilicaulis	*P. decumbens*	*P. decumbens*	IBT 11843	GCA_002072245.1	ALONE
	Fasciculata	*P. camemberti*	*P. camemberti*	FM 013	GCA_000513335.1	X
		P. commune	***P. fuscoglaucum***	FM041	GCA_000576735.1	X
		P. commune	***P. biforme***	FM169	GCA_000577785.1	
		P. crustosum	***P. solitum***	NJ1	GCA_001750005.1	X
		P. freii	*P. freii*	DAOM 242723	GCA_001513925.1	X
		P. nordicum	*P. nordicum*	UASWS BFE487	GCA_000733025.2	X
		P. nordicum	*P. nordicum*	DAOMC 185683	GCA_001278595.1	
		P. polonicum	*P. polonicum*	IBT 4502	GCA_002072265.1	X
		P. polonicum	*P. polonicum*	hy4	GCA_003344595.1	
		P. polonicum	***P. solitum***	RS1	GCA_000952775.2	
		P. solitum	*P. solitum*	IBT 29525	GCA_002072235.1	X
		P. verrucosum	*P. verrucosum*	BFE808	GCA_000970515.2	X
	Lanata-divaricata	*P. brasilianum*	*P. brasilianum*	LaBioMMi 136	GCA_002016555.1	X
		P. brasilianum	*P. brasilianum*	MG11	GCA_001048715.1	
		P. janthinellum	*P. janthinellum*	NCIM1366	GCA_002369805.1	X
		P. oxalicum	*P. oxalicum*	114-2	GCA_000346795.1	X
		P. oxalicum	*P. oxalicum*	JU-A10-T	GCA_000383025.1	
		P. oxalicum	*P. oxalicum*	HP7-1	GCA_001723175.2	
		P. oxalicum	***P. expansum***	YT02	GCA_002072455.1	
		P. subrubescens	*P. subrubescens*	Helsinki	GCA_001908125.1	X

Table S1e

Genus	Section	Actual species	Species of NCBI	Strain/Isolate	Accession NCBI	Note
Penicillium	*Penicillium*	*P. digitatum*	*P. digitatum*	Pd1	GCA_000315645.2	X
		P. digitatum	*P. digitatum*	PHI26	GCA_000315665.1	
		P. digitatum	*P. digitatum*	Pd01-ZJU	GCA_000485865.1	
		P. digitatum	*P. digitatum*	PDC 102	GCA_001307865.1	
		P. expansum	*P. expansum*	NRRL 62431	GCA_000584915.1	X
		P. expansum	*P. expansum*	R19	GCA_000688875.1	
		P. expansum	*P. expansum*	d1	GCA_000769735.1	
		P. expansum	*P. expansum*	MD-8	GCA_000769745.1	
		P. expansum	*P. expansum*	CMP-1	GCA_000769755.1	
		P. expansum	*P. expansum*	T01	GCA_001008385.1	
		P. expansum	*P. expansum*	R21	GCA_001750045.2	
		P. italicum	*P. italicum*	PHI-1	GCA_000769765.1	X
		P. italicum	*P. italicum*	B3	GCA_001008395.1	
		P. italicum	*P. italicum*	GL-Gan1	GCA_002116305.1	
	Robsamsonia	*P. coprophilum*	*P. coprophilum*	IBT 31321	GCA_002072405.1	X
		P. griseofulvum	*P. griseofulvum*	MRI314	GCA_001735785.1	X
		P. griseofulvum	*P. griseofulvum*	PG3	GCA_001561935.1	
		P. vulpinum	*P. vulpinum*	IBT 29486	GCA_002072255.1	X
	Roquefortorum	*P. carneum*	*P. carneum*	LCP05634	GCA_000577495.1	X
		P. paneum	*P. paneum*	FM227	GCA_000577715.1	X
		P. roqueforti	*P. roqueforti*	CECT 2905	GCA_001939915.1	X
		P. roqueforti	*P. roqueforti*	FM164	GCA_000513255.1	
		P. roqueforti	*P. roqueforti*	UASWS P1	GCA_000737485.2	
		P. roqueforti	*P. roqueforti*	JCM 22842	GCA_001599855.1	
	Sclerotiora	*P. sclerotiorum*	*P. sclerotiorum*	113	GCA_001750025.1	ALONE
	N/A	*P. spp.**	*P. spp.*	MA 6036	GCA_003138045.1	ODD

Table S2 Listing of enzyme observations found in all sections of both Ascomycota and Basidiomycota. None of these observations were included in the dendrogram in Fig. 2. The columns A and B refer to occurrence of such enzyme observations in the phyla Ascomycota and Basidiomycota, respectively. The number listed is the percentage of species within the phylum having the specific observation.

Observation	Substrate	A	B
AA1:1.10.3.2	Laccase	99	100
AA3:Unknown	General	98	95
AA9:1.*.*.*	LPMO	94	53
AA11:1.*.*.*	LPMO	100	31
CE4:Unknown		87	72
GH3:3.2.1.21	Cellulose	99	98
GH5:3.2.1.4	Cellulose	90	81
GH7:3.2.1.176	Cellulose	82	55
GH12:3.2.1.151	Xyloglucan	61	14
GH16:2.4.1.*		93	0
GH16:3.2.1.*		93	0
GH16:3.2.1.39		95	73
GH17:Unknown		100	2
GH17:2.4.1.*		40	0
GH18:3.2.1.14	Chitin	98	58
GH31:3.2.1.20	Starch	85	83
GH43:Unknown		87	47
GH43:3.2.1.99	Pectin	71	30
GH72:2.4.1.*		100	84
GH76:3.2.1.101		100	38
GH92:Unknown	Cellulose	79	66
GH132:3.2.1.*		95	0
PL1:4.2.2.10	Pectin	63	14
PL4:4.2.2.23	Pectin	64	45

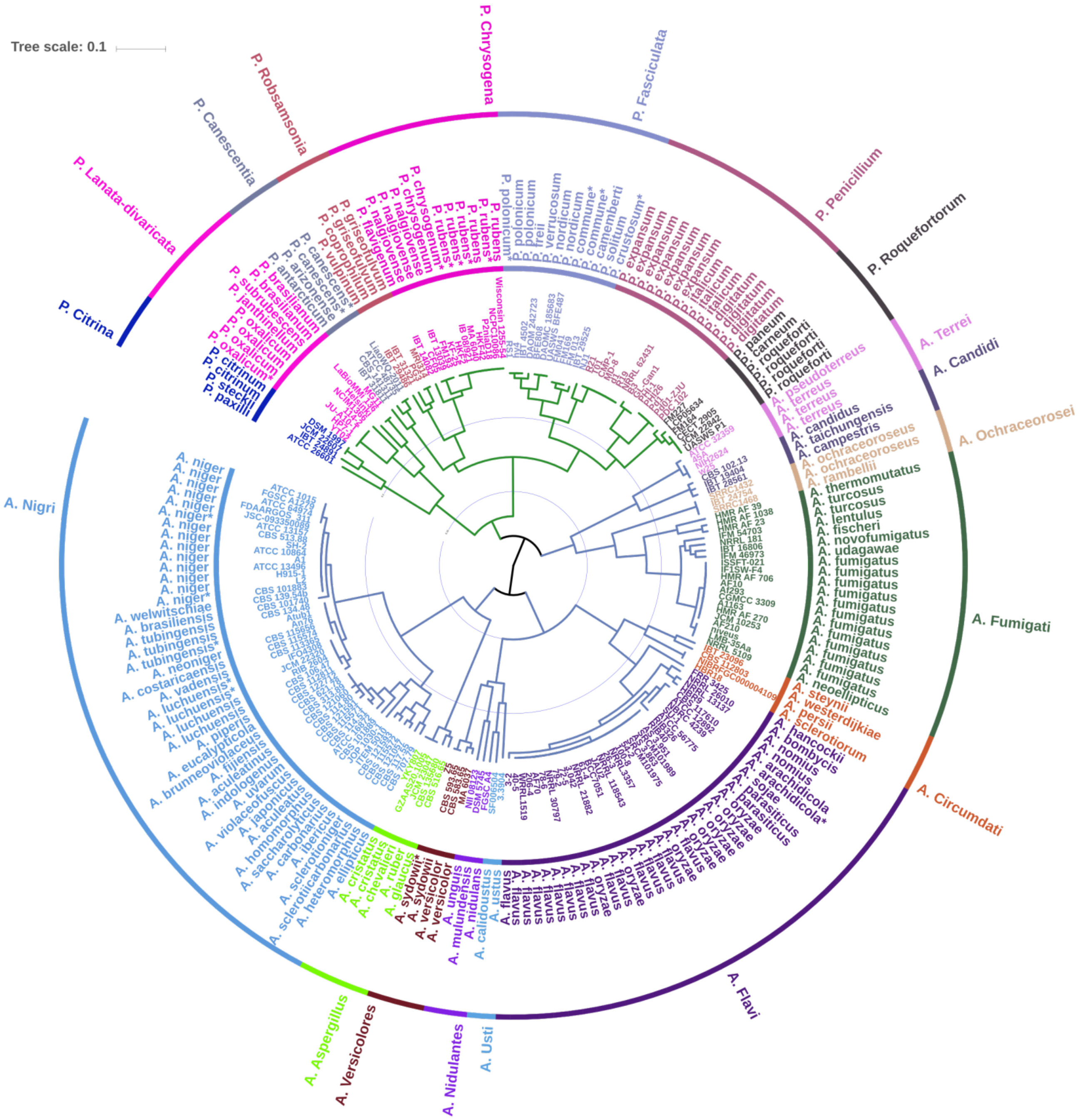

Figure S1 EPR dendrogram including all available strains, which passed filtering of each species within *Aspergillus* and *Penicillium*. The asterisk indicates that the taxonomy of the individual specimens has been revised based on prior phylogenetic assessment. The inner blue ring divides the species into eight groups, including one group of all members of *Circumdati* and *Flavi* now found alone in their own branch.

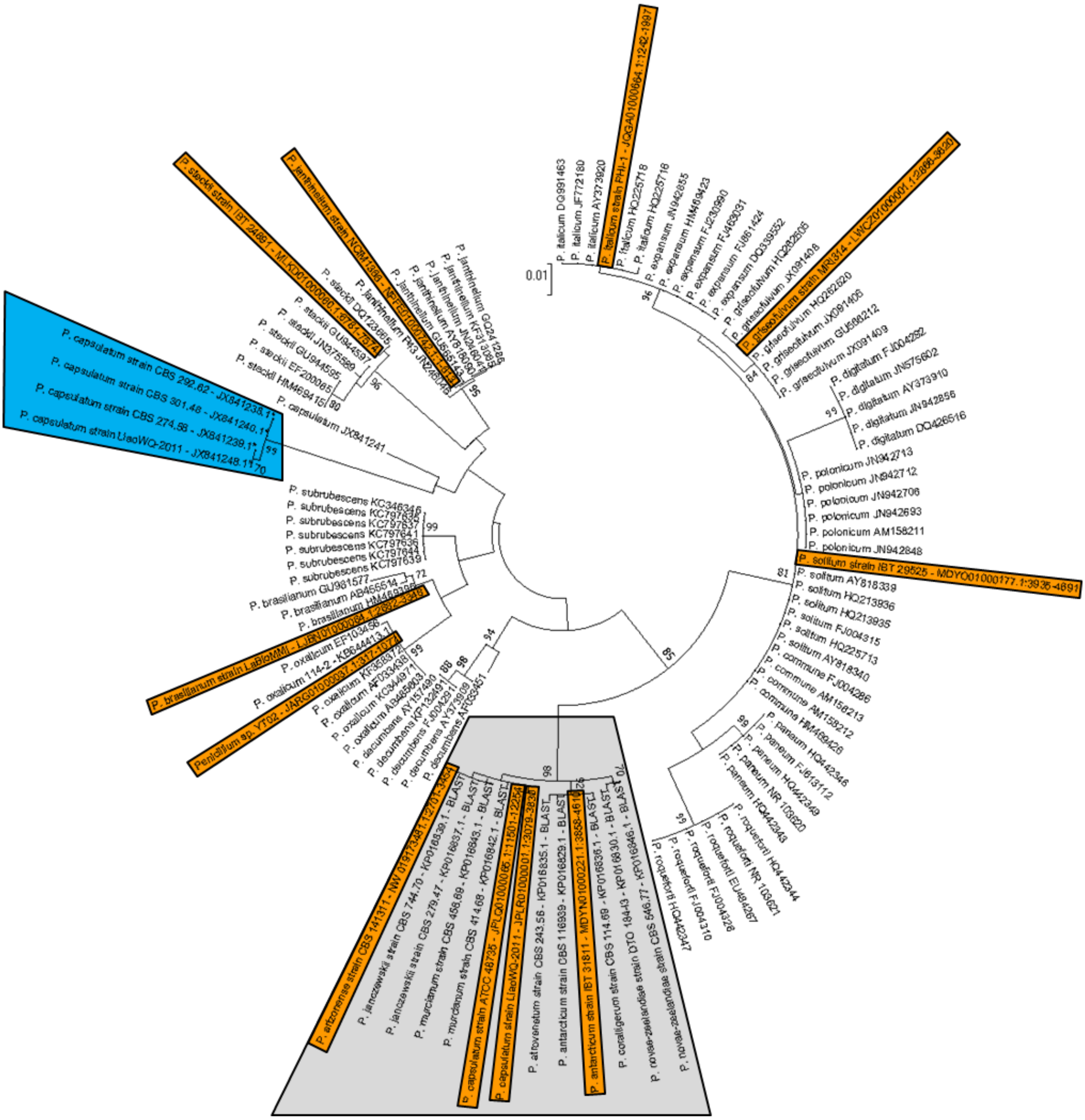

Figure S2 Molecular Phylogenetic analysis by Maximum Likelihood method. The evolutionary history was inferred by using the Maximum Likelihood method based on the Tamura-Nei model. The tree with the highest log likelihood (-1882.14) is shown. Initial tree(s) for the heuristic search were obtained automatically by applying Neighbor-Join and BioNJ algorithms to a matrix of pairwise distances estimated using the Maximum Composite Likelihood (MCL) approach, and then selecting the topology with superior log likelihood value. The tree is drawn to scale, with branch lengths measured in the number of substitutions per site. The analysis involved 107 nucleotide sequences. All positions with less than 99% site coverage were eliminated. That is, less than 1% alignment gaps, missing data, and ambiguous bases were allowed at any position. There were a total of 453 positions in the final dataset. Evolutionary analyses were conducted in MEGA7 and the numbers are bootstrap values of 100 iterations. An asterisk (*) indicates an ITS obtained from the strain based on PCR amplification and sequencing of only the ribosomal internal transcribed spacer region. A suffix BLAST is the top hits found when *Penicillium* strain LiaoWQ-2011, contig JPLR01000001.1, positions 3079-3830 was used as query in a BLAST search. The blue box indicates the ITS barcodes published in the original research paper for the strain of *P. capsulatum* whereas the grey box covered the species belonging to section *P. Canescentia*. The orange box frames the ITS barcodes retrieved from the genomes in the current study placing the two *P. capsulatum* strains within the *P. Canescentia* section.

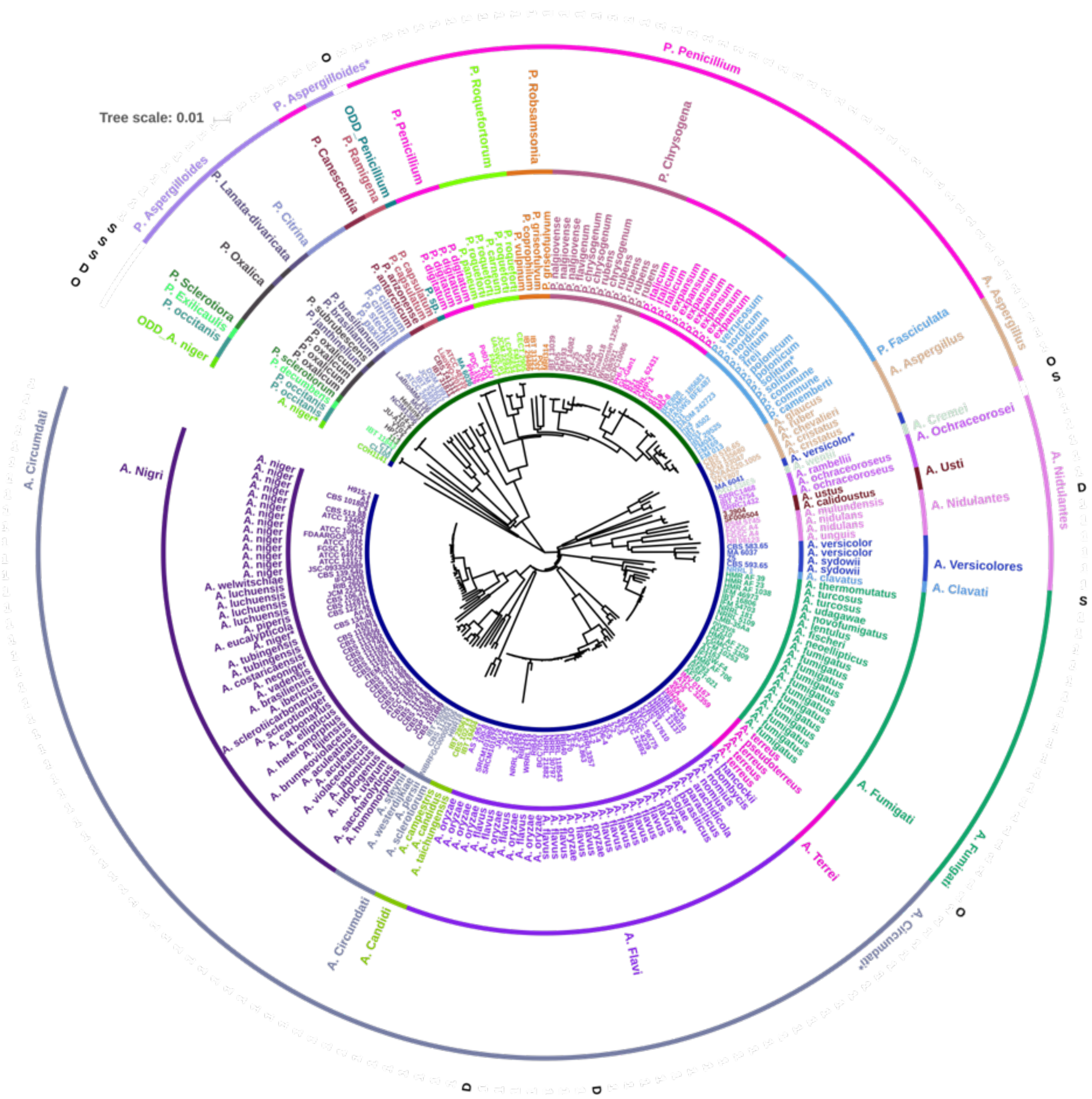

Figure S3 Phylogenetic analysis based on 14 Muscle aligned Tubulin/HSP protein for all available genome assemblies of *Aspergillus* or *Penicillium*. Entities with a the letter D are considered duplicates since the two sequences are available for the same strain and only the newest is considered for further analysis. The letter O indicates that the here presented taxonomical does not agree with the NCBI taxonomy identification and cannot be inferred to any established taxonomy among the current genome assemblies. The letter S indicates that a single genome assembly representing its entire section by only that entity thus disregarded for further analysis. Species names prefixed by an asterisk indicate a probable misidentification of related species.

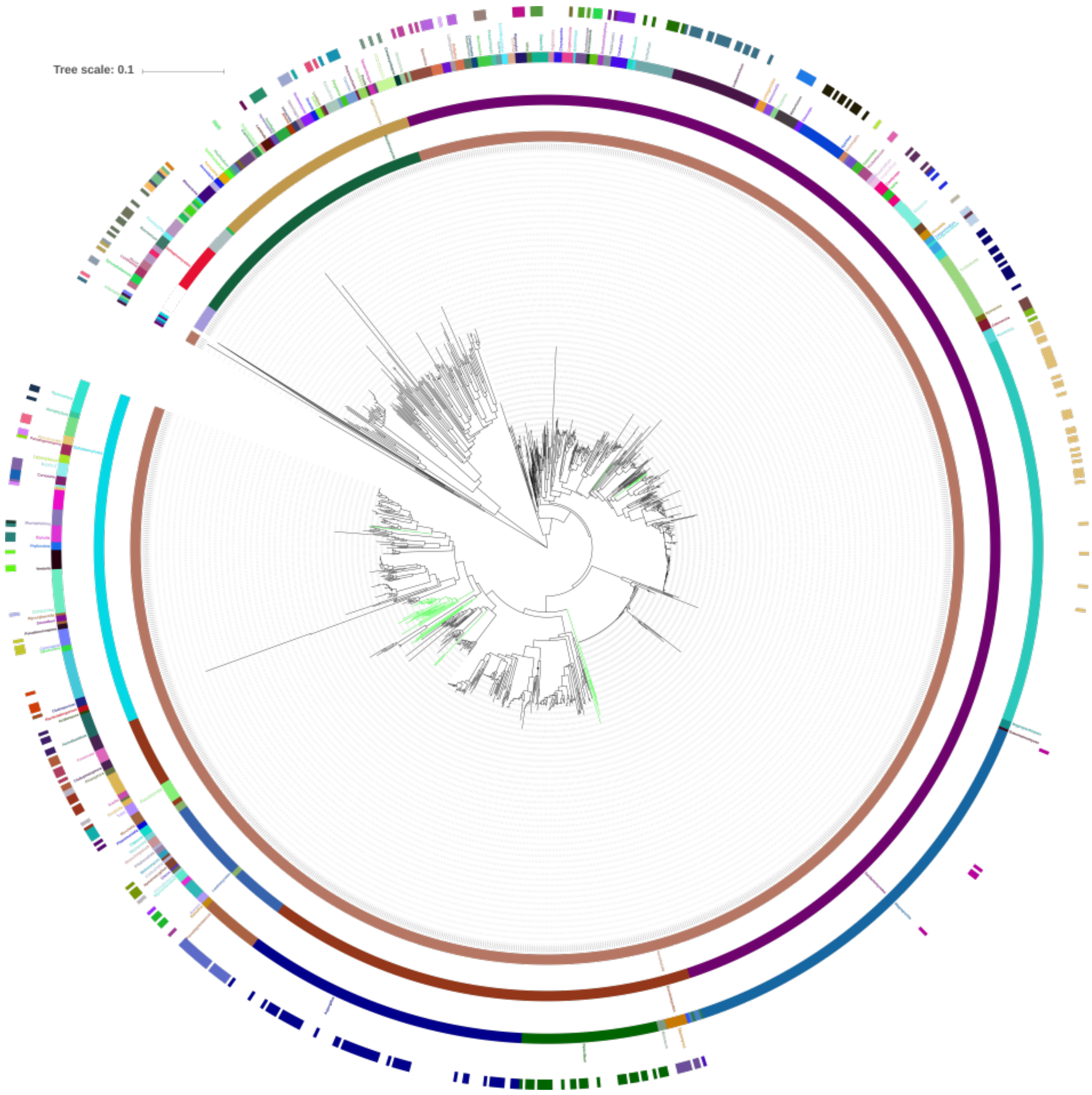

Figure S4 Two HSP combined with two tubulin genes were aligned for 1181 fungal genomes and used to construct a phylogenetic tree based on Maximum Likelihood. The inner ring is phylum, second ring is class, third ring is genus and the outer ring indicates if the entity was included or not prior to this filtering. The green colored branches of the tree indicates removed genome assemblies. See more detailed figure here: https://itol.embl.de/tree/19238139270451537296388. For the plot having representatives from all around the fungal kingdom; genomes leaking barcode genes were initially removed. The four barcodes were aligned and trimmed and concatenated to construction of a phylogenetic tree used for outlier inspection. Genomes placed outside their respective phylum, class or genus were removed.
